# Financial incentives for a healthy life style and disease prevention among older people: a systematic literature review

**DOI:** 10.1186/s12913-016-1517-0

**Published:** 2016-09-05

**Authors:** Marzena Tambor, Milena Pavlova, Stanisława Golinowska, Jelena Arsenijevic, Wim Groot

**Affiliations:** 1 Department of Health Economics and Social Security, Institute of Public Health, Faculty of Health Sciences, Jagiellonian University Collegium Medicum Krakow, Krakow, Poland; 2 Department of Health Services Research, CAPHRI, Maastricht University Medical Center, Faculty of Health, Medicine and Life Sciences, Maastricht University, Maastricht, The Netherlands; 3 Top Institute Evidence-Based Education Research (TIER), Maastricht University, Maastricht, The Netherlands

**Keywords:** Financial incentives, Healthy life style, Disease prevention, Older adults

## Abstract

**Background:**

To motivate people to lead a healthier life and to engage in disease prevention, explicit financial incentives, such as monetary rewards for attaining health-related targets (e.g. smoking cessation, weight loss or increased physical activity) or disincentives for reverting to unhealthy habits, are applied. A review focused on financial incentives for health promotion among older people is lacking. Attention to this group is necessary because older people may respond differently to financial incentives, e.g. because of differences in opportunity costs and health perceptions. To outline how explicit financial incentives for healthy lifestyle and disease prevention work among older persons, this study reviews the recent evidence on this topic.

**Methods:**

We applied the method of systematic literature review and we searched in PUBMED, ECONLIT and COCHRANE LIBRARY for studies focused on explicit financial incentives targeted at older adults to promote health and stimulate primary prevention as well as screening. The publications selected as relevant were analyzed based on directed (relational) content analysis. The results are presented in a narrative manner complemented with an appendix table that describes the study details. We assessed the design of the studies reported in the publications in a qualitative manner. We also checked the quality of our review using the PRISMA 2009 checklist.

**Results:**

We identified 15 studies on the role of explicit financial incentives in changing health-related behavior of older people. They include both, quantitative studies on the effectiveness of financial rewards as well as qualitative studies on the acceptability of financial incentives. The quantitative studies are characterized by a great diversity of designs and provide mixed results on the effects of explicit financial incentives. The results of the qualitative studies indicate limited trust of older people in the use of explicit financial incentives for health promotion and prevention.

**Conclusions:**

More research is needed on the effects of explicit financial incentives for prevention and promotion among older people before their broader use can be recommended. Overall, the design of the financial incentive system may be a crucial element in their acceptability.

**Electronic supplementary material:**

The online version of this article (doi:10.1186/s12913-016-1517-0) contains supplementary material, which is available to authorized users.

## Background

Major health problems affecting today’s societies, such as chronic diseases, are largely preventable and possible to restrain through healthy lifestyle and early detection. However, people sometimes neglect preventive measures and engage in unhealthy habits such as smoking, alcohol abuse or sedentary lifestyle. Thus, policy makers, health insurers and employers all over the world, seek effective approaches to promote a healthy behavior and to stimulate the use of preventive services among the population, as means of reducing health costs and increasing employee’s productivity. These interventions are traditionally targeted towards younger age groups as those who have more years of live ahead and can benefit from prevention activities for longer period of time. Nevertheless, with the ageing of the population, which affects (or soon will affect) various countries all over the world, health promotion for older people is gaining importance as it may bring considerable social benefits.

A variety of mechanisms can be used to change people’s health-related behavior. They range from educational interventions, through subtle guiding of people to make healthier choices (nudging) and more coercive incentives to alter behavior, to paternalistic bans on unhealthy goods [1]. As one of the ways to incentivize a positive health behavior change, financial mechanisms have been applied [2, 3]. For instance, many disease prevention services or lifestyle intervention are provided to consumers free of charge to stimulate their use, while the price of unhealthy products (e.g. tobacco, alcohol) is increased through taxes, which is proven to reduce the consumption of these products [4, 5]. There are also more explicit financial incentives to motivate the change of health-related behavior, such as financial reward for attaining health-related targets (e.g. smoking cessation, weight loss or increased physical activity) or financial penalties for failing to adhere to healthy behaviour.

Economic theory provides the rationale for the application of financial incentives to alter health-related behavior. Due to the present-biased time preferences (myopia, where people place too great a value on current costs and benefits than future ones), people tend to attach lower value to the delayed and uncertain benefits of healthy behavior or preventive services, while they are attracted by the immediate reward of unhealthy habits (e.g. the pleasure of smoking cigarette and alcohol consumption) [6]. Thus, economic theory suggests that financial incentives can be effective as they increase the value of gratification and benefits from healthy behavior or costs of unhealthy habits.

Yet, the evidence on the effectiveness of explicit financial incentives is inconclusive. For instance, a review of studies in the area of tobacco smoking [7] indicates that financial rewards significantly increase smoking cessation. However, the positive effects are frequently not sustainable and tend to dissipate when the financial reward is no longer in place. Similarly, the long-term effects of explicit financial incentives on physical activity and weight loss remain uncertain [8–10]. There is some evidence indicating that explicit incentives may however, work better for less complex behaviors such as attendance to appointment, immunization or screening [2, 11]. The use of explicit financial incentives to modify health-related behavior has also brought about an ethical discussion [12–14]. These incentives are criticized for being coercive or unfair as scarce resources are spent on paying to people who intentionally engage in risky behavior. Moreover, such incentives may interfere in the patient-physician relation and undermine personal responsibilities for own health.

There is even more uncertainty with regard to the use of explicit financial incentives to promote health among older individuals. A number of factors may influence this population group to respond differently to financial incentives than younger groups. On the one hand, the cost of changing behavior can be relatively high for seniors (e.g. engaging in physical activity when having physical limitations and risk of falls) [15, 16]. Also, the benefits from health promotion and health prevention are frequently limited due to a shorter life expectancy at older age. On the other hand, older adults might have more time available (lower opportunity cots) to engage in health-related activities (e.g. walking and physical exercises) than working-age population, or might value health more highly than individuals at the earlier stages of life [16, 17].

In order to outline how explicit financial incentives for healthy lifestyle and disease prevention work among older persons, this study aims to review the recent evidence on this topic. We apply the method of systematic literature review as defined by Grant and Booth (2009) [18]. We focus on explicit financial incentives (rewards and penalties) that aim to promote health and stimulate disease prevention as well as screening. Despite the various recent literature reviews on financial incentives for a health-related behavior change (e.g. [8, 10, 19, 20]), none of these reviews explicitly focused on older adults. Hence, our study fills a gap in our knowledge by providing a comprehensive overview of what is known on the effectiveness and cost-effectiveness of explicit financial incentives on the health behavior of older people. This may facilitate policy makers in their decisions to invest in interventions to modify older people’s behavior. The results of our study also allow us to indicate the need and direction for further research on financial mechanisms to alter health behavior in this population group.

## Methods

To identify relevant literature on the role of financial incentives in health promotion and prevention among older persons, we applied a 4-component search term: ‘health promotion’, ‘incentive’, ‘financial’, ‘elderly’. Possible synonyms, plural forms or different spellings of the terms were also included in the search (see Table 1).

**Table 1:** Chain of keywords used in the literature search

Component	Keywords
Health promotion	“health promotion” OR “promotion” OR “primary prevention” OR “prevention” OR “screening” OR “screenings”
Incentive	“incentive” OR “incentives” OR “motivation” OR “motivations” OR “motivate” OR “stimulus” OR “stimuli” OR “stimulate” OR “reward” OR “rewards” OR “reinforcement” OR “reinforcements”
Financial	“financial” OR “economic” OR “economics” OR “monetary” OR “money” OR “payment” OR “payments” OR “pay” OR “bonus”
Elderly	“elderly” OR “aged” OR “old” OR “senior” OR “seniors”

The exact query used in PubMed: (“aged” [MeSH Terms] OR “aged” [All Fields] OR “elderly” [All Fields] OR “old” [All Fields] OR “senior” [All Fields] OR “seniors” [All Fields]) AND (“health promotion” [MeSH Terms] OR “health promotion” [All Fields] OR “promotion” [All Fields] OR “primary prevention” [MeSH Terms] OR “primary prevention” [All Fields] OR “prevention” [All Fields] OR “screening” [All Fields] OR “screenings” [All Fields]) AND (“motivation” [MeSH Terms] OR “motivation” [All Fields] OR “motivations” [All Fields] OR “motivate” [All Fields] OR “incentives” [All Fields] OR “incentive” [All Fields] OR “stimulus” [All Fields] OR “stimuli” [All Fields] OR “stimulate” [All Fields] OR “reward” [MeSH Terms] OR “reward” [All Fields] OR “rewards” [All Fields] OR “reinforcement” [MeSH Terms] OR “reinforcement” [All Fields] OR “reinforcements” [All Fields]) AND (“economics” [MeSH Terms] OR “economics” [All Fields] OR “economic” [All Fields] OR “financial” [All Fields] OR “payments” [All Fields] OR “payment” [All Fields] OR “pay” [All Fields] OR “monetary” [All Fields] OR “money” [All Fields] OR “bonus” [All Fields])

The search was conducted in November 2015 from the publications published in the last 10 years. The following databases were searched: PUBMED, ECONLIT, COCHRANE LIBRARY. The search in PubMed and Cochrane Library also included MeSH terms. The exact query used in PubMed is presented in Table 1.

To select publications relevant for the review, several inclusion and exclusion criteria were used. Publications were considered relevant if they focused on explicit financial incentives in health promotion, primary prevention or screening among older persons. We defined health promotion and primary prevention as activities that aim to reduce the probability of illness by stimulating a healthy lifestyle and providing services that might decrease the future incident of illness (e.g. vaccination) [21]. We also included studies, which deal with financial incentives to increase participation in screening among older persons (i.e. activities, which aim to detect disease or risk factors for disease among apparently healthy individuals, in this case older individuals). The evidence on incentives to adhere to a drug therapy was not covered by the review.

We included papers on explicit financial incentives targeted at consumers, excluding evidence on motivating health care providers through reimbursement mechanisms to increase provision of preventive services for older population. We also excluded implicit incentives used to remove financial barriers to positive behavior change (e.g. decreasing the price of preventive health services to increase their use) or increase financial barriers to discourage unhealthy behavior (tax on tobacco and alcohol, higher insurance premium for risky health behavior). Thus, we focus on explicit financial incentives, that cover rewards, e.g. cash, gifts or voucher for positive behavior change; and penalties, e.g. losing own money when betting with others on the success in behavior change (deposit contracts/commitment contracts). These incentives could be guaranteed (obtaining reward) or non-guaranteed (the chance of winning reward in lottery). Furthermore, the explicit financial incentive should have aimed to change health behavior, not to increase participation in the clinical research. No limitation with regard to the institutions which provided the incentives, was applied, i.e. papers that presented the programs/initiatives/policies by state, insurers, employers and others were all considered relevant.

With regard to the group of older people, we applied a minimum age limit of 50 years. We primary searched for studies targeted at older population. However, we also looked at studies, which included a broader age category (also younger individuals), if extracting information on older person (50+) was possible. If this was not possible, we included the publication only if the mean age of the study group was at least 60 years.

In addition to this, publications were retained in the review only if they presented an original empirical study (quantitative or qualitative) or a review of empirical studies. Discussion papers, opinion papers and editorials were excluded. There was no limit with regard to the geographical region where the study was conducted, however, we included only English language publications with a full text available and that could be downloaded.

In the first step of the search, the title and abstract of all papers obtained in the search were reviewed to select potentially relevant publications based on the inclusion and exclusion criteria (initial screening). The review of the first 50 titles and abstract was conducted by two independent researchers. Then, the results obtained by the researchers were compared and after assuring high conformity of the results, the review was continued by one researcher. In the second step, the full text of potentially relevant publications was downloaded and screened for their relevance to the study topic by applying the same inclusion and exclusion criteria as those in the initial screening.

The publications selected as relevant were analyzed. We applied the method of directed (relational) content analysis [22], defining five categories (themes) relevant to the review topic: characteristics of the study, characteristics of the study population, behavior targeted by the incentives, characteristics of financial incentives, the outcomes (effectiveness of the incentives or attitudes towards incentives). The data on these categories were extracted from the selected publications, synthesized and presented in a narrative manner complemented with an appendix table that describes the study details.

We assessed the design of the studies reported in the publications in a qualitative manner by reviewing the limitations reported in the discussion sections of the publications as well as based on our own assessment of the design description in the methods section of the publications. We also checked the quality of our review using the PRISMA 2009 checklist (see Additional file 1).

## Results

**Fig. 1:**
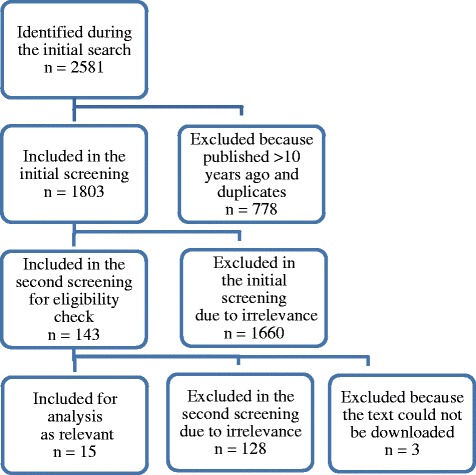
The process of selection of publications for the review

Figure 1 presents the selection process of publications obtained in the search using the chain of keywords presented in Table 1. In total, 2581 publications were identified in the initial search. After applying the year limitation, i.e. publications that appear in the last 10 years (2005/01/14–2015/11/11), 1941 publications remained (891 from PUBMED, 1029 from COCHRANE LIBRARY, 21 from ECONLIT). After removing the duplicates (138), 1803 publications were included in the initial screening. The review of the title and abstract of these publications resulted in 143 potentially relevant publications, including 20 review papers.

During the second screening, the text of the potentially relevant publications was screened applying the same inclusion and exclusion criteria as in the initial screening. This gave 15 relevant (eligible) papers that were analyzed [23–37].

Additional file 2 presents the details of the studies reported in the analyzed publications. Among the 15 studies selected for the review, eight studies [23–25, 27, 29, 32, 34, 35] specifically focus on older people (sample includes only 50 years old or over). There is a great diversity in the socio-demographic characteristics of the participants. There are studies targeted at veterans [26, 27, 37], individuals with specific health problems [27, 33, 37], sedentary adults [24, 25, 32] or those from low-income rural households [29]. In most studies, the sample is dominated by women. This is especially the case of studies for which the recruitment was based on self-selection, i.e. the voluntary responses to the media advertisement [23–25]. The samples in the studies on veterans [26, 27, 37] are however, largely dominated by men.

Almost half of the identified studies (7 out of 15) are from the USA. Three studies are based in Europe, all in Western European countries, i.e. Germany [31], the Netherlands [33] and the UK [36]. The other studies are from Australia [28], Mexico [29], South Africa [30], Canada [34] and Israel [35]. We identified both, quantitative studies on the effectiveness of financial incentives in modifying older adults behavior (*n* = 11), and qualitative studies on the attitudes towards the incentives (*n* = 4).

### Quantitative studies

The review of quantitative studies indicates a great heterogeneity in the design of the studies. We identify six randomized controlled trials [23–28], among them 5 from the USA, with a study period from 1 month [25, 26] to 6 months [23, 24, 27, 28] (including post-intervention fallow-up in some studies) and sample size from 45 participants [24] to 1549 participants [26]. In addition to the randomized controlled trials, there are three non-interventional studies aiming at evaluating the effects of governmental or insurer programs, i.e. Oportunidades program in Mexico [29], Vitality wellness program in South Africa [30], and the preventive bonus program of German Sickness Fund [31]. We also identified two studies [32, 33], which present the quantitative results from stated preference studies on the willingness of older people to participate in the prevention programs with financial incentives.

The behavior most often targeted by the financial incentives is physical activity. The effectiveness of financial incentives in motivating adults to walk more is evaluated in three randomized controlled trials [23–25] (mostly women participating), and also in two stated preference studies [32, 33]. Yet, this incentivized behavior is evaluated differently in the studies, e.g. as number of steps per day or week, minutes of continuous walking per week, days of walking certain number of minutes.

Another frequently analyzed health-related behavior in quantitative studies is screening [26, 28, 30]. One study focuses on the effectiveness of explicit financial incentives to increase immunization rates, evaluating the effects of Oportunidades governmental program in Mexico [29]. In two studies [31, 33], incentives are used to motivate more than one health prevention activity. For example in the German Sickness Fund program [31], physical activity, screening, immunization and check-ups are taken into account when granting financial incentives.

The explicit financial incentives in the quantitative studies reviewed include only positive incentives (rewards). None of the studies presents the research on penalties (deposits). Most of the evaluated incentives are guaranteed rewards [25, 27–33] as oppose to non-guaranteed rewards, such as lottery or raffle [23, 24]. One study looks at both, guaranteed and non-guaranteed rewards [26]. Further, the incentives include largely cash rewards, with only few studies analyzing non-cash rewards, such as shopping voucher [28], discounts for goods [30] or in-kind benefits such as bag, watch etc. [31]. The value of the incentives also differs significantly across the studies with the higher values for non-guaranteed rewards, e.g. participation in $500 raffle for screening completion [26]. Furthermore, financial incentives are combined with other measures to change health-related behavior, such as peer network [23], motivational meetings [24] or information brochure [28].

The findings from the quantitative studies identified in the review, do not give a clear answer to the question on the effectiveness of explicit financial incentives (rewards) in changing consumer health-related behavior. For example, from the three randomized controlled trials on the effects of financial rewards on physical activity, two studies [24, 25] indicate that the incentives are effective in increasing walking among older adults, while one study [23] shows no effect of monetary incentive on meeting walking goals. However, these studies present a great heterogeneity, in terms of duration of the intervention, magnitude and type of the reward, as well as population targeted and sample size. For example, in the study that shows no effects [23], relatively active older adults (*n* = 92) during a 16-week intervention could win weekly monetary reward of max. $200 if they increase their baseline number of daily steps by 50 % in 5 of the past 7 days. On the other hand, two studies which indicate the effectiveness of rewards, specifically focus on inactive older adults, they present a shorter intervention, i.e. 12 weeks [24] and 4 weeks [25], and they are based on a smaller sample size (45 and 51 participants respectively). In the former, participants enter into a lottery with a change of winning up to $100 for each day in a week, during which the recommended number of steps was met [24]. In the latter, the reward is guaranteed i.e. participants receive weekly variable payment (up to $25) depending on the average daily number of aerobic minutes during a week [25].

Similarly, there is no conclusive evidence on the effects of explicit financial incentives on screening participation studied in the randomized controlled trials. For example, the results of one 6-month randomized controlled trial [28] indicate that receiving $25 shopping voucher for attending cardiovascular risk assessment with GP, does not significantly increase screening attendance, while in another 30-day randomised controlled trial [26], the effectiveness of lottery (1 in 10 changes of $50) in increasing the rates of faecal occult blood test completion, was found. Also, we observe mixed results in the studies on the stated willingness to participate in health promotion activities when receiving incentives [32, 33].

On the other hand, non-interventional studies on selected effects of governmental and insurer programs using financial incentives show rather promising results. Namely, in Mexico, a higher immunisation rate was observed among those older individuals who received cash transfer conditional on adherence to various activities, including attendance at a monthly health seminar and compliance to scheduled preventive health check-ups [29]; in South Africa, financial incentives (discount on selected goods) increase the likelihood of colorectal cancer screening (but not prostate and osteoporosis screening) among insured older persons [30], while in Germany older adults in bonus payment program when they receive rewards for participation in various prevention activities, generated significantly lower health care expenditure, leading to cost savings of sickness fund [31]. The German study is the only among the identified studies which presents the economic analysis of the program. All other quantitative studies focus on the effectiveness of financial incentives without analysing cost-effectiveness or cost-benefits of the programs.

As mentioned in the methods section, we assessed the study designs in a qualitative manner by reviewing the study limitations reported in the publications as well as based on our assessment of the study design. Overall, common shortcomings of the randomized controlled trials are a small study sample [23–25] and its non-representativeness of the general populations (e.g. studies include largely highly educated participants with high health status [23, 25], women [23–25], male veterans [26, 27] or patients of a given health care facility [26–28]). The researchers also acknowledge a short duration of these studies (no longer than 6 moths). The main limitation of identified non-interventional studies [29–31] is the possibility of unmeasured confounders and difficulties to investigate the causal effects of incentive on the outcomes measured. Two stated preference studies on the willingness of older people to participate in the prevention programs with financial incentives [32, 33] suffer mainly from their hypothetical nature, i.e. respondents’ hypothetical statements might not be reflected in real-life situations.

### Qualitative studies

Our review also included four qualitative studies on the attitudes of older individuals towards financial incentives. In three of these studies [34–36], data are collected through focus group discussions and in one study this is done through semi-structured interviews [37]. In two studies, focus group discussions are narrowed down to a specific group of older adults and their health-related behaviour, i.e. physical activity among cardiac rehabilitation patients [34] and adherence to colorectal cancer screening among the individuals eligible for screening [35]. In both studies, the views on positive (rewards) incentives are studied. The third study based on focus group discussions [36], explores the opinions of the general older adults population on both, positive (rewards) and negative (penalties) incentives to modify health behavior. Semi-structured interviews are, on the other hand, conducted among the participants of a randomized controlled trial (veterans) to collect data on their attitudes towards financial reward used in this trial [37].

All studies reveal the lack of trust among older adults about explicit financial incentives. The main concerns identified in the studies are: immorality and unfairness towards those who take care of their own health and might have to finance rewards for those who engage in risky health behavior [34–37], perception of incentives as bribery [34–36], questionable effectiveness and waste of scarce resources [34, 36], risk of abusing the scheme [36], harm to the physician-patient relationship or undermining individual autonomy and intrinsic motivation [35, 37].

Some forms of incentives seem to be more accepted than others. For example, respondents showed a preference for positive rewards rather than negative penalties or deposits [36], for in-kind (shopping or gym vouchers) rather than cash incentives [34, 36], for guaranteed reward rather than lottery [36], privately sponsored rather than government funded incentives [34]. In the opinion of older people, providing more tailored and meaningful incentives [34, 36] can prove better results. The respondents also acknowledge the importance of the size of incentives which in their opinion, should be sufficient for the incentive to be effective [34, 36, 37]. The acceptability of financial incentives is greater if they prove to be effective [36]. In one study [36], education and peer support were mentioned as being more appropriate strategy than financial incentives, to change people's behavior using public resources.

The limitations of the identified qualitative studies include: restriction of the results to a specific population group - only veterans [37] or individuals with middle and low socio-economic status [35], small number of focus groups [34], limited openness of the respondents to discuss this issue [36] and typical for focus group discussions - the possibility of moderator bias. Moreover, it should be acknowledged that the studies were conducted in various countries with a specific health system environment, including Canada [34], Israel [35], the UK [36] and the USA [37], which may have affected the opinion of the respondents.

## Discussion

The aim of this paper was to review the recent evidence on the role of financial incentives in encouraging healthy lifestyle and disease prevention among older persons. We reviewed English-language papers published in the last 10 years, selecting studies on the use of explicit financial incentives (financial rewards or penalties) for changing older adults behavior.

Although financial incentives are increasingly being used to motivate people to modify their health-related behavior and improve health outcomes, our results indicate that there is little attention for the evaluation of the role of financial incentives for health prevention among older adults. We identified only 15 relevant studies: six randomized controlled trails, three non-interventional quantitative studies evaluating the effects of government/insurer programs, two quantitative stated preference studies and four qualitative studies on the attitudes towards financial incentives. Seven studies are from the USA, while other parts of the world are represented only by few studies. The lack of interest in analyzing the effects of financial incentives on health-related behavior among older adults can be explained by the traditional focus of prevention and health promotion on younger individuals. However, more research on the effective measures to promote health among older individuals is expected as this group becomes a substantial part of countries’ population all over the world.

A broad range of health-related behaviors has been analyzed in the studies, including attendance to immunization or screening, and more complex behaviors such as physical activity. The most attention is being paid to the use of financial incentives in promoting physical activity (walking). This is not surprising as physical activity has an important role in healthy ageing, namely preventing chronic diseases and improving the quality of life of older adults [38–40]. All studies identified are on rewards rather than penalties (deposit/commitment contracts). The research on the effectiveness of penalties for health promotion and prevention among younger population groups yields mixed results [7, 41–43], with promising findings from some recent studies [44, 45]. Given that these programs might be less costly to implement than reward-based programs (as they do not require funds for rewards), their use to change health behaviour among older people merits more research.

The randomized controlled trials identified in our review, do not give a clear answer to the question on the effectiveness of explicit financial incentives in modifying health-related behavior of older adults. Mixed results are provided in both, the studies on the effects of rewards on physical activity and in the trials on less complex behavior change (attendance to vaccination or screening), which could be expected to show greater effectiveness of incentives [2, 12]. The heterogeneity of the results may be partly attributed to the differences in design of the programs, e.g. type of financial reward (guaranteed vs. non-guaranteed), magnitude of reward, immediacy and frequency of reward, feasibility of achieving rewarded goal. These elements have been identified in the literature on financial incentives as potential determinants of the program’s success [11, 12, 46, 47]. However, the small number of identified studies and their diversity do not allow us to conclude on the most effective design of the incentives in promoting health among older adults.

Drawing conclusions on the effectiveness of the financial incentives is also hindered by the uncertain quality of the evidence. The common limitations of the identified randomized controlled trials are the short study duration (max. 6 months and for some studies only 4 weeks) and, in some studies, lack of post-intervention follow up. These study characteristics will be crucial for concluding on the usefulness of explicit financial incentives for health promotion. The studies for the general population provide indications that the effectiveness of financial incentives decreases with the time of intervention and the behavioural change is not sustainable when the incentive is not in place any more [2, 3, 11, 12]. The explanation of this phenomenon is provided in the literature on people’s motivation [48, 49]. Financial incentives constitute an example of extrinsic motivation, which induces behavioural change by increasing the immediate benefits associated with the behaviour. However, financial reward does not increase the persons’ intrinsic motivation (engaging in activity for its inherent satisfaction rather than pressure or reward) and hence, the positive effects are vanished after the reward is ceased [3].

Another important limitation of the included intervention studies is a small non-representative character of the samples, which limits external validity of these studies. For example, studies on physical activity include participants who voluntary responded to the advertisement, which resulted in recruiting mostly women, and in some studies, individuals with better health and socio-economic status who may be less sensitive to small financial incentives, as indicated in the literature on the topic [11, 12, 50]. Other identified studies focus on specific population groups, such as US veterans who are found to have a greater health awareness and appreciation of health care benefits they receive, which may make financial incentives less necessary and effective in this group than in the general population of older adults [37].

Promising results are provided by non-interventional studies analyzing the effects of existing government and insurer programs with the use of financial rewards, i.e. Mexican Oportunidades cash-transfer program on immunisation rate among older individuals from low-income rural households, Vitality program in South Africa on the adherence to health screening test recommendations, and the preventive bonus program in German health insurance on cost savings. Positive results of these programs have been indicated also in other studies which are not the subject of this review, e.g. [51–54]. Yet, it should be noted that these studies possess several limitations. Most importantly, it is unclear to what extent the observed effects can be attributed to the financial incentive rather than other (confounding) factors. Hence, further evaluation of these programs, with a greater attention given to older population groups, should be undertaken.

While the results of the qualitative studies on the effectiveness of explicit financial incentives are not conclusive, the studies on the attitudes towards these incentives confirm the concerns about the use of financial incentives in changing health-related behavior. The common argument emerging from the studies is of ethical nature. Paying to those who intentionally engage in risky behavior such as smoking or abuse drinking, is considered immoral and unfair towards individuals perusing a healthy lifestyle. Thus, there is a limited acceptability of such programs to be financed from restrained public resources. The results indicate that non-government funded programs such as employer-sponsored incentives (workplace wellness programs) might find more approval among the public. Such programs have been increasingly applied in the USA [11, 55, 56], but they are less common in European countries where employers to a lesser extent bear the cost of health care. The benefits from workplace programs are derived mainly by working population, yet, this increasingly includes older individuals. Thus, such programs should be of interest to the stakeholders in ageing policy.

Other relevant issue discussed in identified qualitative studies is the possibility of adverse effects of financial incentives, such as undermining individual responsibility for health. The research on the intrinsic motivation has shown that extrinsic rewards can reduce intrinsic motivation [49, 57]. People may become reliant on receiving a reward for behaviour and if the reward is ceased, the behaviour is seen as less worthwhile. Some researchers also argue that financial reward for one behaviour might increase persons expectations to be financially rewarded also for other behaviours [13, 58]. The extent to which these adverse effects are evident when applying financial rewards for health promotion is largely unknown [48]. However, there is a need for caution when using financial incentives, in order to reduce the risk of their unintended consequences.

Although our review was systematic and we took care to assure its quality (see Additional file 1), there are some limitations, which need to be acknowledged. Most importantly, the search and the analysis were largely conducted by one researcher. We limited the risk of selection bias by performing the initial part of the review by two independent researchers, and assuring high conformity of their results. There is also a risk that we might have missed some relevant evidence as we included in the review only English-language studies, and only published studies. There could be some relevant studies still under review. Further, the quality of the evidence was evaluated in a qualitative manner without applying a standardized protocol.

## Conclusions

The results of this review indicate that, although there is a significant body of evidence on the effectiveness of financial incentives in promoting healthy lifestyle and disease prevention, few studies (mostly from USA) focus on older adults or present specific results for this population group. The heterogeneity of quantitative studies identified in this review, and their limitations, do not allow for clear conclusions on the effectiveness of financial incentives. However, the qualitative studies indicate low acceptability of these mechanisms among older individuals. This calls for a careful design of health promotion programs with the use of financial incentives to account for the preferences of older people.

Our results indicate a need for further research and a more thorough investigation of the effectiveness and cost-effectiveness of different financial incentives (both positive and negative) in changing older people’s behavior. The limitations reported in the studies, such as a small and non-representative sample size, or a short study duration, should be addressed. Given the variety of ways to change people’s behavior, it is worthwhile to devote more attention to the application of financial incentives together with non-financial tools from behavioural science such as nudges, peer support, etc. The study on how to align financial incentives targeted at consumers with supplier-side incentives, may also be a valuable contribution to the research and health promotion policies.

## Additional files

Additional file 1: Results of the assessment of our review using the PRISMA 2009 Checklist. (PDF 328 kb) Additional file 2: Details of quantitative and qualitative studies included in the review. (PDF 252 kb)
