# Supplementary material for: Financial incentives for a healthy life style and disease prevention among older people: a systematic literature review
Source: BMC Health Serv Res. 2016 Sep 5;16(Suppl 5):426. doi: 10.1186/s12913-016-1517-0 (PMC5016732; doi:10.1186/s12913-016-1517-0)
Supplement: Additional file 2: — Details of quantitative and qualitative studies included in the review. (PDF 252 kb) [file 12913_2016_1517_MOESM2_ESM.pdf]

Additional file 2. Details of quantitative and qualitative studies included in the review

	Study (author/s and year)	Participants	Behaviour targeted by the incentives	Financial incentives	Results/outcomes	Study characteristics	Study limitations
	**Quantitative studies**						
1	Kullgren JT, Harkins KA, Bellamy SL, Gonzales A, Tao Y, Zhu J, Volpp KG, Asch DA, Heisler M, Karlawish J (2014)	Older adults (≥65) recruited from a general population who voluntary responded to the advertisement (n= 92)<br><br>Mean age: 72y<br>Sex: M (30%)	Walking - meeting walking goals (increase daily steps by 50%) in 5 of the past 7 days	- A lottery with a 3 in 10 chance of winning $50 and a 3 in 100 chance of winning $200 every week if walking goals were met in at least 5 of the past 7 days (expected value for the lottery - approx. $21)<br><br>Nonfinancial incentive:<br>-peer network through an online message board<br><br>Control group: participants receiving weekly feedback on the achievement of walking goals<br><br>All participants receive pedometer	**NO significant results on the effectiveness of financial incentives:**<br><br>Financial incentives, and financial incentives combined with peer network, did not result in a significantly higher mean proportion of days walking goals were met during the 16-week intervention. Also peer networking alone was ineffective.<br><br>During the post-intervention 8-week follow-up period, there was no significant decrease in mean proportion of days walking goals were met. Still, there was no significant difference between the intervention group and the control group in mean proportion of days walking goals were met.	Randomized controlled trial (16 weeks + 8-week post-intervention follow up)<br><br>Country: USA, 2011-2012	- Small study sample<br>- Lack of representativeness of the general population (mostly women, highly educated, with high health status)<br>- Risk of volunteer bias (more motivated to exercise)<br>- Fairly short study duration
2	Petry NM, Andrade LF, Barry D, Byrne S (2013)	Sedentary (walking less than 6000 steps per day) older adults (55-75y) from a general population who voluntary responded to the advertisement (n= 45)<br><br>Mean age: 63y<br>Sex: M (8-24%)	Walking – meeting waking goals (recommended number of steps) per day:<br>1st week≥6000 steps per day<br>2nd week≥ 800 steps per day<br>3-12 week≥ 10 000 steps per day	A lottery with changes to win $1-$100, for each day the target number of steps was met + motivational weekly meetings + $5 gift car for each week of participation + $20 for participation in evaluation at the end of the intervention<br><br>Control group: motivational weekly meetings + $5 gift car for each week of participation + $20 for participation in evaluation at the end of the intervention	**Significant results on the effectiveness of financial incentives:**<br><br>Financial incentives resulted in a significantly higher number of days walking goal were met.<br><br>In 12-week post intervention follow-up, there was reduction in number of steps, yet there was still significant difference between intervention group and control group.	Randomized controlled trial (12 weeks +12-week post intervention follow-up)<br><br>Country: USA	- Small study sample<br>- Lack of representativeness of the general population (mostly women)<br>- Risk of volunteer bias (more motivated to exercise)<br>- Fairly short study duration

	Study (author/s and year)	Participants	Behaviour targeted by the incentives	Financial incentives	Results/outcomes	Study characteristics	Study limitations
3	Finkelstein EA, Brown DS, Brown DR, Buchner DM (2008)	Sedentary (exercising < 2 h per week) older adults (50+) recruited from the Raleigh–Durham area of North Carolina who voluntary responded to the advertisement (n= 51)<br><br>Mean age: 60y<br>Sex: M (25%)	Walking - aerobic minutes defined as 10+ minutes of continuous walking or jogging.	Variable incentive payment depending on average daily aerobic minutes during each of the 4weeks of the study (with four thresholds, up to $25 if average aerobics minutes per day ≥ 40). Additionally fix payment of $50 for participation<br><br>Control group: fixed payment of $75 for participation	**Significant results on the effectiveness of financial incentives:**<br><br>Financial incentives resulted in significantly higher number of aerobic minutes (16 more aerobic minutes per day after controlling for sociodemographic characteristics).	Randomized controlled trial (4 weeks)<br><br>Country: USA, 2007	- Small study sample<br>- Lack of representativeness of the general population (mostly women, highly educated)<br>- Risk of volunteer bias (more motivated to exercise)<br>- Short study duration and lack of follow-up
4	Kullgren JT, Dicks TN, Fu X, Richardson D, Tzanis GL, Tobi M, Marcus SC (2014)	Veterans - primary care patients of clinics in Philadelphia<br>(n= 1 549)<br><br>Mean age: 61-63y<br>Sex: M (99-100%)	Screening – completion of faecal occult blood test within 30 days	- Receiving $5, or $10, or $15 for test completion (I stage)<br>- Receiving $5, or 1 in 10 change of $50, or entry into $500 raffle for test completion (II stage)<br><br>Control group: usual care, no financial incentive to complete the test	**Mixed results on the effectiveness of financial incentive:**<br><br>Fix guaranteed reward of $5, $10, or $15 as well as entry into $500 raffle did not increase the rates of test completion.<br>Lottery – 1 in 10 changes of $50 was effective in increasing the rates of test completion.	Randomized controlled trial (30 days)<br><br>Country: USA, 2012	- Lack of representativeness of the general population (male veterans, patients of a given health care facility)<br>- Short study duration and lack of follow-up
5	Long JA, Jahnle EC, Richardson DM, Loewenstein G, Volpp KG (2012)	African-American Veterans at the Philadelphia Medical Center with poor diabetes control (n= 118)<br><br>Age range: 50-70y (mean age: 59-60y)<br>Sex: M (90-100%)	Reduction of haemoglobin A_{1C} (HbA_{1C}) levels after 6 months	Receiving $100 if decreasing HbA_{1c} level by 1% and $200 is decreasing it by 2% or to an HbA_{1c} level of 6.5%.<br><br>Nonfinancial incentive: peer mentoring<br><br>Control group: usual care, no mentoring and no incentives	**NO significant results on the effectiveness of financial incentives**:<br><br>The decrease HbA1c levels in a group with financial incentives was not statistically significant.<br><br>Peer mentors had a statistically significant effect on improvement of glucose control.	Randomized controlled trial (6 months)<br><br>Country: USA, 2009-2010	- Lack of representativeness of the general population (male veterans, patients of a given health care facility)

	Study (author/s and year)	Participants	Behaviour targeted by the incentives	Financial incentives	Results/outcomes	Study characteristics	Study limitations
6	Stocks N, Allan J, Frank O, Williams S, Ryan P (2012)	Patients aged 40–74, without diagnosed cardiovascular disease (n= 301) from two Adelaide urban general practices<br><br>Sex: M (41%)<br>Older adults (+60) in the sample: 57%	Screening - cardiovascular risk assessment with GP	Receiving $25 shopping voucher for attending no cost cardiovascular risk assessment with GP within 6 months of the invitation date + information brochure<br><br>Control group: invitation letter to attend cardiovascular risk assessment without financial incentive + information brochure	**NO significant results on the effectiveness of financial incentives**:<br><br>Monetary incentive did not lead to a statistically significant increase in screening attendance.<br><br>Attendance in both groups (with and without financial incentive) was low.	Randomized controlled trial (6 months)<br><br>Country: Australia	- Lack of representativeness of the general population (patients of a given health care facility)
7	Salinas-Rodriguez A, Manrique-Espinoza BS (2013)	Older adults (≥65) from low-income households from 741 rural communities of 13 Mexican states participating in governmental program Oportunidades. (n= 4 628)<br><br>Mean age: 74y<br>Sex: M (52%)	Vaccination - self-reported immunization status regarding tetanus, influenza and pneumococcal vaccines	Cash transfer conditional on adherence to activities, such as: a) school enrolment of children age 6–16; b) attendance by an adult at a monthly health seminar and, c) compliance by all family members to schedule preventive health check-ups (for older adults every 6 months)	**Significant results on the effectiveness of financial incentives:**<br><br>The proportion of older people vaccinated was significantly higher among those receiving cash transfers (Oportunidades participants) than among those not covered by the Oportunidades program.	Cross-sectional study, based on 2007 Oportunidades Evaluation Survey<br><br>Country: Mexico	- Risk of unmeasured confounders<br>- Risk of self-reporting bias<br>- Limited to low-income adults
8	Adonis L, Basu D, Luiz J (2014)	Random sample of health insurance members from a single insurer (n= 170 471), including:<br><br>- Eligible insured persons for colorectal cancer screening (adults ≥50y, n= 4 963)<br><br>- Eligible insured persons for prostate-specific antigen screening (males ≥50y, n=107)<br><br>- Eligible insured persons for Osteoporosis screening (females ≥60y, n= 46)	Adherence to health screening test recommendations (colorectal cancer screening, prostate-specific antigen screening, osteoporosis screening)	Discounts at network stores for goods like electronic equipment, books, airline flights, car rentals, and holidays	**Mixed results on the effectiveness of financial incentive:**<br><br>Receiving incentives (belonging to Vitality wellness program) increases the likelihood of colorectal cancer screening but not prostate and osteoporosis screening.	Cross-sectional study for 2007-2011<br><br>Country: South Africa	- Risk of unmeasured confounders<br>- Lack of representativeness of the general population

	Study (author/s and year)	Participants	Behaviour targeted by the incentives	Financial incentives	Results/outcomes	Study characteristics	Study limitations
9	Stock S, Stollenwerk B, Klever-Deichert G, Redaelli M, Büscher G, Graf C, Möhlendick K, Mai J, Gerber A, Lüngen M et al. (2008)	Members of the single largest nationally operating sickness fund in Germany (n= 140 858)<br><br>Mean age: 41<br>Sex: M (26%)<br><br>Older adults (+50) in the sample: 44%	Screening, immunization and check-ups, physical activity	Bonus payment (in-kind benefits or €30) for earning 500 credit points in one year or two successive years. Points are granted for participation in prevention activities (more than one activity)	**Significant results on the effectiveness of financial incentives:**<br><br>Adults aged 50-74:<br>Those who participated in preventive bonus program have significantly lower hospital cost, medicine cost and additional treatment cost (physical therapy, massage) than those who did not participate in the program.<br><br>Adults aged 75+:<br>Those who participated in the program have significantly lower hospital cost, medicine cost but not additional treatment cost than those who did not participate in the program.	Prospective controlled cohort study, January 2004-December 2005<br><br>Country: Germany	- Risk of unmeasured confounders<br>- Risk of overestimation of the effect of the intervention due to exclusion of patients who enrolled but did not start the intervention
10	Brown DS, Finkelstein EA, Brown DR, Buchner DM, Johnson FR (2009)	Inactive adults aged ≥50 (n= 501) from general population<br>Sex: M (47%)	Physical activity - participation in the walking program	Weekly cash payment as one of the attributes of the walking program in a survey on willingness to participate in the program	**Significant results on the effectiveness of financial incentives:**<br><br>Independent of other program attributes, modest financial incentives increase the likelihood of program participation by sedentary older adults<br>e.g. offering an incentive of $9 in cash per week ($468 per year) increases predicted participation in a walking programme (3days/week 30 min outside of a formal group setting) by 31%.	Stated preferences study - Conjoint analysis<br><br>Country: USA, 2006	- Risk of hypothetical bias

	Study (author/s and year)	Participants	Behaviour targeted by the incentives	Financial incentives	Results/outcomes	Study characteristics	Study limitations
11	Wanders JO, Veldwijk J, de Wit GA, Hart HE, van Gils PF, Lambooij MS (2014)	Diabetes mellitus type 2 (DM2) patients from four health care centers in a geographically defined area of the Netherlands (n=206)<br><br>Mean age: 62y<br>Sex: M (54%)	Participation in lifestyle intervention program - diet, physical activity	Cash payment for completing the program as one of the attributes of lifestyle program in a survey on willingness to participate in the program	**NO significant results on the effectiveness of financial incentives**:<br><br>Negative association between financial reward and willingness to participate in the program, i.e. an increase in the cash payment decreases willingness to participate in the program.	Stated preferences study - Discrete choice experiment<br><br>Country: Netherlands	- Risk of hypothetical bias<br>- Lack of representativeness of the general population
	**Qualitative studies**						
12	Mitchell MS, Goodman JM, Alter DA, Oh PI, Faulkner GE (2014)	Canadian cardiac rehabilitation patients (n= 15) recruited from the Toronto Rehabilitation Institute<br><br>Age range: 54-84y (mean age: 65y)<br>Sex: M (67%)	Physical activity	Positive financial incentives (rewards)	**Low acceptability and negative attitude toward financial incentives:**<br><br>Financial incentives are considered unfair, burden to public health system, unnecessary or a waste of limited resources.<br><br>Financial incentives are accepted under certain conditions.<br>Preferences for privately sponsored (not government funded), non-cash incentives (e.g. grocery or gym vouchers) and with the possibility of donating them to charity.<br><br>In the opinion of participants, incentives to be effective should be of sufficient size.<br>Incentives are perceived to be more effective for lower socio-economic groups.	Three focus group discussions of 5–6 participants March-April 2013<br><br>Country: Canada	- Limited to patients of a given health care facility<br>- Small number of focus groups

	Study (author/s and year)	Participants	Behaviour targeted by the incentives	Financial incentives	Results/outcomes	Study characteristics	Study limitations
13	Hagoel L, Rennert G, Feder-Bubis P (2015)	Eligible for colorectal cancer screening individuals (aged 50–68y) from two urban, middle and low socio-economic status primary care clinics in a Northern city in Israel (n= 24).<br><br>Sex: M (42%)	Screening - adherence to colorectal cancer screening	Positive financial incentives (rewards)	**Low acceptability and negative attitude toward financial incentives:**<br><br>Incentives are considered unsuitable for solving screening difficulties (on rational and moral grounds). They are seen as undermining a doctor–patient relationship and patient individual autonomy.	Six focus group discussions November 2009 - February 2010<br><br>Country: Israel	- Limited to individuals from middle and low socio-economic status, patients of a given health care facility
14	Giles EL, Sniehotta FF, McColl E, Adams J (2015)	Adults (18+) from North East England (n= 74)<br><br>Older adults (+60) in the sample: approx. 50%<br><br>Sex: M (53%)	Health behavior	Positive (rewards) and negative (penalties) financial incentives	**Low acceptability and negative attitude toward financial incentives:**<br><br>Financial incentives are accepted under certain conditions:<br>if they are fair; if they are closely monitored and evaluated; if they are shown to be effective and cost-effective (only than financed from public resources).<br>Preference for positive rewards rather than penalties, for rewarding healthy behaviour rather than giving up unhealthy behaviour, and for shopping vouchers rather than cash incentives (though some individuals would prefer cash incentives as more flexible in use).<br>More acceptable recipients of incentives are pregnant women, nursing mothers and those on lower incomes.<br><br>Education and peer support are perceived as more appropriate than financial incentives.<br><br>Older respondents did not differ in their views from younger respondents.	Eight focus group discussions of 7-12 participants November 2013 - January 2014<br><br>Country: UK	- Limited openness of the respondents for discussion<br>- The lack of knowledge on the effectiveness of financial incentives may have contributed to the negative attitudes towards them

	Study (author/s and year)	Participants	Behaviour targeted by the incentives	Financial incentives	Results/outcomes	Study characteristics	Study limitations
15	Reisinger HS, Brackett RH, Buzza CD, Paez MB, Gourley R, Weg MW, Christensen AJ, Kaboli PJ (2011)	Hypertensive veterans participating in an intervention to promote guideline-consistent therapy from Veterans Affairs primary care clinics (n= 54).<br><br>Mean age: 65y<br>Sex: M (98%)	Visiting primary care provider and talking about hypertension and initiating clinical practice guideline-concordant therapy (thiazide diuretics) for hypertension	Receiving $20	**Low acceptability and negative attitude toward financial incentives:**<br><br>Most participants stated the incentive did not influence their decision to initiate a discussion with their provider. This was confirmed in randomized control trial which showed modest not significant effect of incentive.<br><br>Incentive was perceived as unnecessary to promote discussion with provider, inappropriate to accept or too low to influence behaviour.<br><br>Some acceptability of incentives if they are proven to be effective.	Semi-structured interviews<br><br>Country: USA	- Limited to specific group – veterans of a given health care facility
